# Modular Tissue‐Engineered Motor Units Featuring Spinal Motor Neurons Innervating Self‐Assembled Myofiber Bundles

**DOI:** 10.1002/mabi.202500660

**Published:** 2026-08-13

**Authors:** Melanie C. Hilman, Elizabeth N. Krizman, Foteini Mourkioti, Suradip Das, D. Kacy Cullen

**Affiliations:** ^1^ Center For Brain Injury & Repair Department of Neurosurgery Perelman School of Medicine University of Pennsylvania Philadelphia Pennsylvania USA; ^2^ Center For Neurotrauma Neurodegeneration & Restoration Corporal Michael Crescenz Veterans Affairs Medical Center Philadelphia Pennsylvania USA; ^3^ Department of Bioengineering School of Engineering and Applied Science University of Pennsylvania Philadelphia Pennsylvania USA; ^4^ Department of Orthopaedic Surgery Perelman School of Medicine University of Pennsylvania Philadelphia Pennsylvania USA; ^5^ Department of Cell and Developmental Biology Perelman School of Medicine University of Pennsylvania Philadelphia Pennsylvania USA; ^6^ Institute For Regenerative Medicine Musculoskeletal Program Perelman School of Medicine University of Pennsylvania Philadelphia Pennsylvania USA

**Keywords:** biomaterial scaffold, innervation, myofiber, neuromuscular junction, skeletal muscle, tissue engineering

## Abstract

A motor unit is the functional unit of muscle contraction, consisting of a population of skeletal muscle fibers innervated by axon terminals from a motor neuron. Tissue engineering strategies are being pursued to treat neuromuscular injuries by mimicking aspects of native myofascicular architecture; however, the critical role of innervation in myofiber development is often overlooked. Our group previously developed a pre‐innervated tissue‐engineered muscle on nanofiber sheets, demonstrating that innervation facilitated myofiber maturation and function in vitro. The current study builds on this framework to biofabricate pre‐innervated three‐dimensional (3D) bundles of individual myofibers that more closely replicate in vivo architecture. Specifically, we established a methodology to generate centimeter‐scale Tissue Engineered Motor Units (TEMUs) comprising aligned myofiber bundles within a collagenous hydrogel and innervated by axons projecting from discrete population(s) of spinal motor neurons. A custom‐built polydimethylsiloxane micro‐scale channel system facilitated the alignment and self‐assembly of myoblasts. The presence of aggregated motor neurons and axonal integration significantly enhanced myofiber maturation and contractility compared to non‐innervated controls. We also evaluated the effects of media constituents on myofiber maturation, as assessed by myocyte fusion and sarcomere formation. Importantly, this TEMU biofabrication protocol is fully scalable, generating modular myofiber bundles at least 8 cm in length that can be aligned in parallel to achieve large‐scale myofiber macro‐bundles. TEMUs address key challenges in muscle tissue engineering by providing a 3D biofidelic platform to study the role of innervation in muscle development and function in vitro, as well as an implantable composite tissue to facilitate muscle replacement after severe trauma.

## Graphical abstract

The current study devised a methodology to biofabricate pre‐innervated, three‐dimensional bundled myofibers, as shown in this confocal micrograph depicting immunolabeled axons (red) and a multinucleated (blue) myofiber with well‐defined sarcomeres (green).

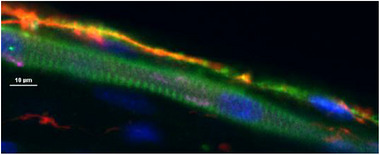

## 1 Introduction

Innervation plays a key role in muscle development, function, and regeneration [1, 2]. For instance, during development, motor axon terminals couple with nascent myofibers and secrete neural agrin, which facilitates the formation and stabilization of the neuromuscular apparatus. Also, axon‐mediated activation of these neuromuscular junctions (NMJs) further promotes maturation and functionality of myofibers. In addition, the presence or absence of innervation critically influences the muscle's response to trauma. For instance, severe injuries like volumetric muscle loss (VML) are often accompanied by motor axotomy, which can severely limit functional recovery [3]. Indeed, despite the post‐injury activation of regenerative muscle stem cells, known as satellite cells, myogenesis in denervated muscle is abortive and fails to produce normal muscle fibers [4]. Thus, the indispensable role of innervation in muscle development and regeneration suggests that incorporating neural inputs during the biofabrication of engineered skeletal muscle tissue could be highly beneficial. The resulting neuromuscular tissue may be useful for various applications, including biofidelic testbeds to study muscle development, function, and disease biology in vitro, or as implantable substrates for muscle replacement following injury in vivo.

The critical role of both three‐dimensionality and innervation in myofiber maturation has been borne out through a growing body of studies using a range of neuromuscular culture systems. For instance, 3D bundles of myofibers encased in hydrogel have long been shown to be associated with longer culture times [5], increased myotube size [6], and increased AchR clustering [7] compared to 2D cultures. Motor neuron‐myocyte coculture studies were performed over many years to better understand cellular mechanisms underlying development and function of NMJs as well as implications in disease and injury [8]. Recently, endothelial cells were grown concurrently in neuron‐myocyte cultures, validating that tri‐culture is possible [9]. Also, 3D coculture systems featuring human stem cell‐derived motor neurons and skeletal myocytes within an extracellular matrix (ECM) have been used to further our understanding of the neuromuscular apparatus [10, 11]. Such 3D neuromuscular cultures facilitate myocyte differentiation as well as the formation of mature and functional NMJs that enable spatiotemporal control of muscle contraction [7, 12]. These engineered systems have been used to study mechanisms of NMJ formation, neurodegenerative diseases like amyotrophic lateral sclerosis and myasthenia gravis, and bioactuator control [7, 13, 14]. Of note, micropatterned ECM has been shown to produce increased fusion of myotubes, larger acetylcholine receptors, increased expression of NMJ genes, and increased myofiber contractility as compared to non‐patterned 3D cultures [15]. This demonstrates that topographical cues influence neuromuscular cultures and should be considered during the biofabrication process.

As such, the field has increasingly recognized the importance of innervation in muscle tissue engineering. Currently, optogenetics [13, 16], neurochemical transmission [17, 18], and myoelectric activation [19, 20] are some of the modes of neuromuscular stimulation to activate muscle contraction. In a novel approach, our group previously fabricated aligned neuromuscular microtissue and demonstrated the efficacy of pre‐innervated tissue‐engineered muscle in maintaining neuromuscular health following VML [21]. However, despite considerable progress in musculoskeletal tissue engineering and 3D neuromuscular cultures, significant challenges remain, particularly in developing scalable engineering solutions that retain biomimetic microarchitecture. For example, while decellularized tissues can achieve bulk restoration, they fail to provide the cellular‐level topographical and biochemical cues necessary for effective regeneration [22]. By and large, muscle tissue engineering strategies, such as nanofibrillar scaffolds populated with exogenous cells to mimic the native microarchitecture [21, 23, 24, 25, 26], are challenging to translate to human‐scale defect sizes.

To address these limitations, there is a critical need for a tissue‐engineered approach that can recapitulate the fascicular architecture and cellular niche of skeletal muscle‐motor units. Accordingly, the current study describes the development of a novel composite engineered microtissue comprising three‐dimensional (3D) centimeter‐scale aligned bundles of myocytes within a collagen hydrogel and innervated by discrete populations of spinal motor neurons, collectively termed tissue‐engineered motor units (TEMUs). We validated a novel biofabrication scheme and optimized culture conditions and media formulation to enhance myocyte fusion, myofiber maturation, and neuromuscular junction (NMJ) formation over time. Importantly, the TEMU biofabrication is fully scalable, capable of producing modular myofiber bundles up to 8 cm in length – with longer lengths readily achievable – that can be aligned in parallel to form large‐scale myofiber macro‐bundles. This engineered tissue builds upon our previous tissue‐engineered innervated muscle models by transitioning from planar nanofiber‐supported constructs to a fully of a 3D extracellular matrix‐based architecture that more closely recapitulates native neuromuscular organization while enabling scalable and modular tissue assembly. As such, it provides a biofidelic in vitro platform for investigating the critical role of innervation in muscle development, function, and injury/disease, and may also serve as an implantable composite tissue to promote re‐innervation and functional recovery of host and graft muscle after severe trauma.

## 2 Methods

All procedures were approved by the Institutional Animal Care and Use Committees at the University of Pennsylvania and the Corporal Michael Crescenz Veterans Affairs Medical Center and adhered to the guidelines set forth in the NIH Public Health Service Policy on Humane Care and Use of Laboratory Animals (2015).

### 2.1 Cell Isolation and Culture Techniques

Commercially available C2C12 mouse skeletal myoblasts were cultured (ATCC CRL‐1772) and primary spinal motor neurons following previous procedures as briefly described below [21].

### 2.2 Spinal Motor Neurons

Cultures of rat spinal motor neurons were dissociated from embryonic spinal cords. Before dissociation, all tissue harvest was done on frozen granite block. Spinal cords were dissected from embryonic day 16 (E16) Sprague‐Dawley (Charles River) rats. The mother rat was euthanized with CO_2_ asphyxiation followed by decapitation. Post decapitation and tail snipping of the embryo, the spinal cord was extracted from the dorsal side and placed in HBSS (Hanks’ Balanced Salt Solution, Invitrogen 14175079). The meninges and dorsal root ganglion were stripped away. Cords were trypsinized with 20 µL of 2.5% 10X Trypsin (Gibco 15090046) in 1 mL of Leibovitz's L‐15 solution (Life Technologies Inc. 11415064), subsequently triturated in DNAse (1 mg/mL Roche Diagnostics 10104159001) and 4% Bovine Serum Albumin (Sigma A4161‐5G) in a base L‐15 solution. After a 10 min centrifugation at 280 × *g* at 22°C, the remaining pellet was resuspended, in Opti‐prep (Abcam ab286850), and centrifuged once again at 520 x g for 15 min at 4°C. The cells were extracted from the interface and resuspended in motor neuron plating media with established growth factors: 37 ng/mL hydrocortisone, 2.2 µg/mL isobutylmethylxanthine, 10 ng/mL BDNF, 10 ng/mL CNTF, 10 ng/mL CT‐1, 10 ng/mL GDNF, 2% B‐27, 20 ng/mL NGF, 20 µM mitotic inhibitors, 2 mM L‐glutamine, 417 ng/mL forskolin, 1 mM sodium pyruvate, 0.1 mM β‐ mercaptoethanol, 2.5 g/L glucose in a neurobasal solution to make complete motor neuron plating media [21, 27].

To create motor neuron aggregates, dissociated cells were plated in pyramid wells [28, 29]. These wells were made of PDMS and formed in a hollow, inverted pyramid shape, with the cells gathering at the tip of the pyramid. A cell suspension of 2.5 × 10^4^ dissociated motor neurons in a volume of 15 µl was added to each pyramid, and centrifuged at 1500 RPM for 5 min. The wells were then flooded with motor neuron plating media and incubated for 24 h to allow the aggregates to grow and form.

### 2.3 Culturing C2C12 Mouse Skeletal Myoblasts

C2C12 myoblasts (Abcam, were cultured and maintained in growth media composed of 20% Fetal Bovine Serum (Sigma F0926‐100ML), 1% Penicillin‐Streptomycin (ThermoFisher 15070063), 79% Dulbecco's Modified Eagle Medium/Nutrient Mixture F12 (Invitrogen 11330032). Upon reaching 80% confluency, myoblasts were detached from the tissue culture flask with 0.25% Trypsin‐EDTA (ThermoFisher 25200056). Growth media was added, and the cell suspension was centrifuged at 300 g for 8 min. The cell pellet was resuspended in growth media such that the cell concentration was 6.5 × 10^6^ cells/mL. Each 1 cm engineered microtissue consisted of 32 500 myocytes and was cultured in growth media.

### 2.4 Forming Cellular Extracellular Matrix (ECM) Solution

The ECM solution is 5% resuspended cell solution, 70% media, and 25% 4 mg/mL collagen (Advanced Biomatrix 5153‐1Kit) to yield a final concentration of 1.3 × 10^6^ cells/mL of myocytes and 1 mg/mL collagen suspended in skeletal myocyte growth media. The homogenous solution is kept on ice and 20 uL of solution is deposited into each 1 cm PDMS microchannel. For the longer microtissues, solution was added to fill the respective channels: 40 uL in 2 cm channels, 80 uL in 4 cm channels, and 160 uL in 8 cm channels. Motor neuron aggregates are placed in the reservoirs immediately after ECM solution is plated and the combined microtissues gel for 15–30 min. Once the microtissues were gelled, media was added. There was a particular media composition paradigm that is explained in Section 2.8.

### 2.5 Biofabrication of Engineered Tissue

Engineered cellular tissue consists of the proper ECM protein constituents for the cell type in use, the optimal concentration of ECM with the cell type, and a scaffolding that enables controlled cell behavior or alignment. The fabrication protocol is detailed below.

### 2.6 Designing 3D Channels

The 3D negative mold was designed using the computer‐aided design software SolidWorks and intended to be placed in a standard 6‐well plate. Channels were dimensioned to be 0.08 cm × 0.15 cm × 1.00 cm (width x height x length). The radius and the depth of the reservoirs were both 0.15 cm (Figure S1). For replicative value, the design included eight channels in one mold. For the 2 and 4 cm microtissues, channels were shaped to fit within the parameters of 100 mm petri dishes; the design for the 8 cm microtissue used a 150 mm petri dish. The negative mold was printed in resin. To make the positive mold, polydimethylsiloxane (PDMS), which consists of a curing agent and an elastomer, was poured into the negative mold and cured in a 50°C oven overnight. After the PDMS mold was removed, it underwent an autoclave cycle and once placed into a 6‐well plate, it was UV sterilized for 30 min.

### 2.7 Cell Culture in 3‐D Extracellular Matrix Using a PDMS Mold as an Exterior Scaffold

The experiment is illustrated in Figure 1. Liquid polydimethylsiloxane (PDMS) was poured into a custom‐built (Figure S1) inverted 3D printed channel molds to harden and form a biocompatible, autoclavable environment to culture cells (Figure 1A). One day prior to the experiment, spinal cord motor neurons were dissociated and left overnight to aggregate in pyramid‐shaped wells [28, 30] (Figure 1B). Myocytes were passaged and combined with ECM solution from Section 2.4 and that with the motor neuron aggregates were (Figure 1D) plated in the PDMS microchannels with neuron aggregates at the endpoints and myocyte+ECM along the complete channel. The tissue was cultured for 7 or 21 days in different cellular configurations (a) **T**issue **E**ngineered **M**uscle, (b) **T**issue **E**ngineered **M**otor **U**nit‐uni with one motor neuron aggregate at one endpoint, and (c) **T**issue **E**ngineered **M**otor **U**nit‐bi with two motor neuron aggregates, one at each endpoint.

**FIGURE 1:**
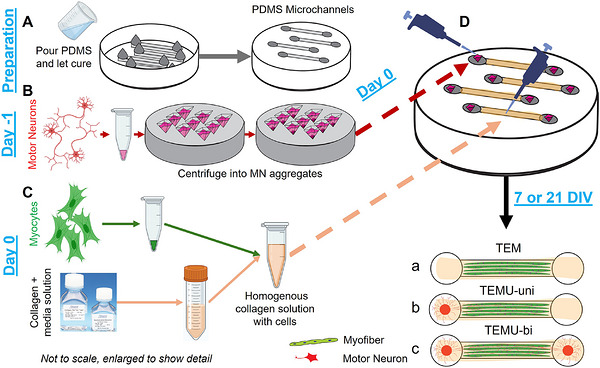
Custom fabrication of tissue engineered muscle and motor units. (A) Formation of mold using PDMS. (B) Aggregation of dissociated motor neurons the day prior to plating. (C) Combining myocytes with ECM into a homogeneous solution. (D) Plating the cellular ECM solution with motor neuron aggregates on either end to form (a) Tissue Engineered Muscle, (b) Tissue Engineered Motor Unit‐uni, (c) Tissue Engineered Motor Unit‐bi.

### 2.8 Optimizing Culture Media by Varying the Presence of a Mitotic Inhibitor

During the first four days in culture, or the growth phase, media consisted of 50% myocyte growth media as described and 50% motor neuron media as described. For the remaining days in culture the media consisted of 50% motor neuron media and 50% myocyte differentiation media composed of 2% Normal Horse Serum (Fisher 16050130), 1% Pen‐Strep, 97% DMEM‐F12 maintained in an incubator at 5% CO_2_, 37°C, and 19.2% O_2_ and sufficient humidity [21, 31]. The motor neuron media had 12 constituents in a neurobasal solution, one was a mitotic inhibitor [32], which could affect myocyte growth. As such, three different media combinations were investigated: mitotic inhibitor (1) present in growth and differentiation medias (Media +/+), (2) present in only differentiation media (Media −/+), or (3) completely absent (Media −/−). Refer to Figure 6 for visual aid.

### 2.9 Removing the Microtissue Pre‐ and Post‐Fixation

To terminate the culture, engineered microtissues that were immunoassayed for confocal imaging and constructs imaged using SEM were fixed in place in the PDMS channels they were cultured in. The microtissues were then carefully removed from the channels using forceps.

### 2.10 Scanning Electron Microscopy (SEM)

Samples were fixed with 2.5% glutaraldehyde in 0.1 M sodium cacodylate buffer (EMS 15960) at a pH 7.4 for 1 h and dehydrated before taken to the SEM core facility at the Singh Center for Nanotechnology (Quanta 600 FEG ESEM) and imaged. A low‐vacuum SEM was used and allows samples to be viewed without requiring a gold sputter‐coating; this avoids a buildup of charge at the sample surface [33].

### 2.11 Immunohistochemistry

At 7 or 21 DIV, the samples were fixed for 35 min in 4% paraformaldehyde (EMS, 15710), washed 3 times with 1x DPBS (Dulbecco's Phosphate‐Buffered Saline, Gibco 14190‐136), and permeabilized in 0.3% Triton‐X100 (Sigma 9036‐19‐5) + 4% Normal Horse Serum (NHS, Fisher 16050130) for 60 min. All subsequent antibody solutions were prepared in 4% NHS. This project called for two staining protocols. First, motor neurons were stained using β‐tubulin III or Tuj1 (1:200, Abcam ab18207) and choline acetyltransferase, the enzyme that synthesizes acetylcholine, was stained with ChAT (1:200, Abcam ab18736) and both antibodies were incubated overnight in 4°C to be subsequently paired with donkey‐anti‐rabbit 568 fluorescent antibody (1:500, Fisher A10042) and donkey‐anti‐sheep 647 fluorescent antibody (1:500, Abcam ab150179) respectively incubated at room temperature for 2 hr. F‐actin was stained with AlexaFluor‐488‐phalloidin (1:400, Invitrogen A12379) and the acetyl‐choline receptors concentrated at neuromuscular junctions were stained with AlexaFluor‐647‐conjugated bungarotoxin (1:250, Invitrogen B35450), both antibodies incubated for 2 hrs in room temperature. The second protocol was the following: motor neurons were stained using neuronal marker neurofilament heavy polypeptide or NF200 (1:200, Abcam ab8135) and myocytes were stained with sarcomeric α‐actinin (1:200, Sigma A7732) incubated overnight in 4°C to be subsequently paired with donkey‐anti‐rabbit 568 fluorescent antibody (1:500, Fisher A10042) and Donkey‐anti‐mouse 488 fluorescent antibody (1:500, Invitrogen A‐21202) respectively and incubated at room temperature for 2 h. The acetylcholine receptors were stained with AlexaFluor‐647‐conjugated bungarotoxin (1:250, Invitrogen B35450) and incubated for 2 h in room temperature. All included samples were stained with Hoechst‐33342 (Invitrogen‐H370) as a DNA marker. Images were acquired using a Nikon Eclipse TI A1RSI laser scanning confocal microscope.

### 2.12 Cryosectioning and the Cross‐Section

The engineered microtissues and naïve muscles were embedded in OCT, cryopreserved, sectioned, and stained. First, freshly harvested whole tissue, either engineered microtissues or naïve tissue, were fixed in 4% paraformaldehyde (EMS 15710), submerged in 20% sucrose in 1X phosphate‐buffered saline (PBS, pH 7.4) for density equilibration, embedded in OCT, and frozen. The engineered muscles were cryosectioned axially (20 µm) while the naïve muscles were cryosectioned longitudinally (20 µm). Prior to staining, sections were washed three times in 1X PBS, blocked and permeabilized in 4% normal horse serum (Sigma G6767) with 0.3% Triton X‐100 (Sigma T8787) in 1X PBS for 1 h. All subsequent steps were performed using blocking solution for antibody dilutions. For staining of engineered muscle, samples were incubated with β‐tubulin III or Tuj1 (1:200, Abcam ab18207, rabbit host) which targets motor neurons and anti‐NeuN (1:200, Sigma ABN91, chicken host) which targets neuronal nuclei overnight at 4 °C followed by AlexaFluor‐568 antibody (1:500, Invitrogen A10042, donkey‐anti‐rabbit) and AlexaFluor‐647 antibody (1:500, Jackson ImmunoResearch 703‐605‐155, donkey‐anti‐chicken) respectively and AlexaFluor‐488‐phalloidin (1:400, Invitrogen A12379) which stains for F‐actin in skeletal myocytes and motor neurons, for 2  h at room temperature. Images were acquired using a Nikon Eclipse TI A1RSI laser scanning confocal microscope.

### 2.13 Data Collection Techniques

The *length* was measured by finding the displacement between the furthest points on a myocyte/myofiber. The *width* was determined by the thickest section in the myofiber. *Aspect ratio* was calculated by dividing the myofiber length by the width*. Myocyte angle orientation* (Figure S2D) was measured with respect to the macro directional orientation of the cylindrical engineered microtissue. The *fusion index* was gathered with the following rationale: myofibrils with three or more nuclei were tallied up and divided by the total number of skeletal myocyte nuclei.

$$\begin{array}{ccc} & & \text{Myocyte Fusion Index}\\ & & =\frac{\text{Number of nuclei in myofibers with more than three nuclei}}{\text{total number of nuclei present in myocytes}} \end{array}$$

The overlap of the Hoechst staining with Phalloidin confirmed the nuclei in each myocyte.

The distance between each sarcomere fiber was measured as *sarcomere length*. The proportion of microtissues with sarcomeres was calculated by dividing the number of myofibers with sarcomeres by the total counted myofibers in a single microtissue.

### 2.14 Data Analysis and Statistical Validation

Data was processed and analyzed using GraphPad PRISM software. All data was collected from three regions of interest with *n* > 3 for each TEMU variant and for each media variant. When comparing cell composition/organization and time in culture, data were analyzed by two‐way analysis of variance (ANOVA) testing. The same test was used when comparing media variations across morphological metrics. Significance was taken at p  ≤  0.05 (*), *p*  ≤  0.01 (**), and p  ≤  0.001 (***). Data were displayed as mean ± standard deviation. Histograms were generated in MATLAB utilizing the entire dataset of individual cell values across at least 3 regions of interest. These data were compared using the Kolmogorov–Smirnov distribution test, considering TEM as a control group and otherwise compared among all groups, whether it was across cell configuration, media type, or days in culture.

## 3 Results and Discussion

A motor unit is the smallest functional unit of muscle contraction, consisting of a population of skeletal muscle fibers innervated by a motor neuron's axon terminals. During development, muscle and nerve grow concurrently and subsequently form neuromuscular junctions at their points of contact. Motor neurons secrete agrin which facilitates the clustering of acetylcholine receptors on skeletal muscle as targets for innervating motor neurons [34]. Over the duration of development, muscles grow, and sarcomeres generate as myocytes fuse and lengthen [35]. The presence of sarcomeres indicates the capacity for muscles to contract over a calcium gradient which is triggered by acetylcholine binding to acetylcholine receptors [36, 37]. Using tissue‐engineering strategies, such as isolating skeletal muscle and neuronal cell populations and leveraging the structural integrity and self‐alignment of extracellular matrix (ECM), we fabricated a Tissue Engineered Motor Unit using a custom‐built apparatus. The custom‐built 3D PDMS mold served as a scaffold for the ECM to gel and form long channeled tissue engineered muscle (Figure 1). The design underwent multiple iterations before the final structure was selected (Figure S1A), with dimensions remaining within the bounds of macro‐scale tissue engineering (Figure S1B, C). From construction to endpoint, the removable cellular microtissues were observed and characterized using various microscopy and histological techniques.

### 3.1 Immunocytochemistry to Visualize Cellular Architecture under Confocal Microscopy

Engineered microtissues were generated in three distinct cellular configurations, as illustrated in the schematic (Figure 2A). A monoculture of self‐aligned skeletal myocytes–referred to as Tissue Engineered Muscle (TEM, Figure 2A,B)–served as the foundation for the fabrication strategy (Figure 1), which was subsequently adapted to include additional cell types such as motor neurons. Aggregated motor neuron populations were added to the endpoints of the column‐like muscle constructs, either on one end—Tissue Engineered Motor Unit‐uni (TEMU‐uni, Figures 2A,C)—or on both ends—Tissue Engineered Motor Unit‐bi (TEMU‐bi, Figure 2A,D). Cell viability was maintained over 7 or 21 days in culture, as depicted by immunofluorescent staining for myocytes (green, phalloidin), neurons (red, Tuj1), and an enzyme that synthesizes acetylcholine named choline acetyltransferase (far red, ChAT). These initial microtissue configurations were cultured in Media +/+ and further developed as described in Section 2.8 and Section 3.5.

**FIGURE 2:**
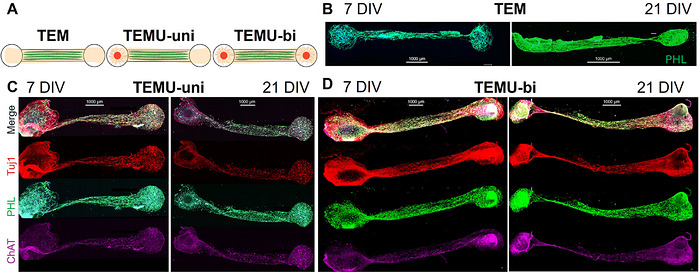
Representative immunofluorescent images of whole length engineered microtissues with endpoints and length. (A) A schematic accompanies all configurations at the respective locations as indicated on the images. The images show myocytes (phalloidin, green), motor neurons (Tuj1 or β‐tubulin III, red), choline acetyltransferase (ChAT, far red), and nuclei (Hoechst) for the three cellular configurations TEM (A,B), TEMU‐uni (A,C), and TEMU‐bi (A,D) at 7 and 21 DIV. Scale bar is 1000 µm for whole length microtissues.

The first generation of this tissue‐engineered muscle/motor units derived from a previous protocol for proliferating and differentiating myocytes in a monoculture for 7 days and then in co‐culture with embryonic spinal cord motor neurons thereafter [9, 21, 23]. Proof‐of‐concept was realized and characterized by combining the protocols together, culturing myocytes and motor neurons co‐currently. In this system, the ratio of myocyte media to motor neuron media was 1:1 and therefore total concentrations of all media elements were halved. Variability in the entire microtissue length was attributed to its elastic range [38], flexibility [37], and potential collapse of collagen fibers [39]. Phalloidin fluorescent antibody stained for actin non‐specific to muscle cells, suggesting that more than one cell population is expressed in the green‐fluorescent signal. On the contrary, β tubulin III served as a neuron‐specific marker. While some neurons migrated away from the neuron aggregates, axons also projected outward into the channel containing myocytes. The positive ChAT expression, which targets the enzyme that produces acetylcholine, indicated the production of the neurotransmitter that activates muscle contraction. The successful co‐culture and biomimetic alignment of myocytes demonstrated by this proof‐of‐concept immunostaining provided the foundation for subsequent investigation on myofiber growth and functional innervation.

### 3.2 Ultrastructural Analyses to Observe Exterior Microtissue Morphology

To observe the exterior microarchitecture of the TEMU and its variants, we performed scanning electron microscopy on the groups of the three cellular configurations (Figure 3). At the endpoints, where motor neuron aggregates reside in the TEMU‐uni and TEMU‐bi groups, the microarchitecture appeared disordered and clustered (Figure 3A). The area where the aggregate leads into the channel body, called the neuro‐muscle transition, suggests axons projecting out of the motor neuron aggregate along the striations of the self‐aligned collagen fibers (Figure 3B). Along the channel region, there are self‐aligned striated fibers (Figure 3C) that extend along the length of the channel (Figure 3D). This nanoscale surface visualization technique confirmed a fibrous, aligned morphology.

**FIGURE 3:**
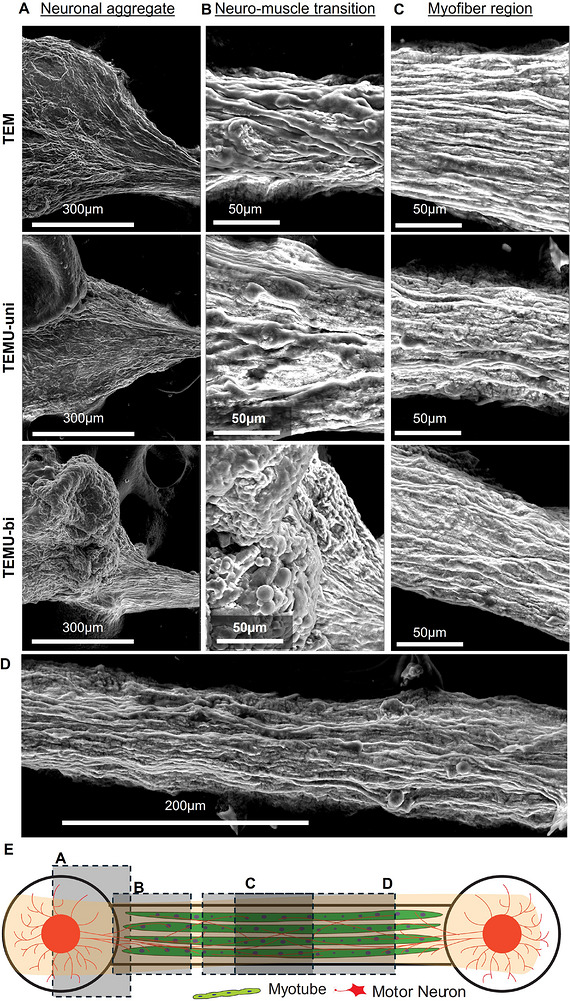
Exterior microarchitecture of engineered microtissues shown by representative scanning electron microscopy images of TEM, TEMU‐uni, and TEMU‐bi. (A) Zoomed‐out images of the neuronal aggregate or the endpoint of the engineered microtissue (with no neuron), (B) zoomed‐in images of the region adjacent to the endpoint region called the neuro‐muscle transition, and (C) images taken from an arbitrary point along the main body of the microtissue. (D) A longer image of the length of the microtissue. (E) All locations are depicted in a schematic.

The electron microscopy images confirm elongated, self‐aligned striations on the microtissues’ exteriors. Collagen and myofibers are known to align in similar patterns [40, 41, 42] as exemplified in Figure 3. Collagen fibers are known to assemble into triple‐helices based on the chemical and spatial structure of the monomers, forming long fibrous structures [43]. Likewise, myocytes fuse to form long anisotropic myofibers. Ultimately, collagen's capacity for anisotropic extension and self‐alignment guides myocyte fusion and a uniform alignment [44]. Therefore, although collagen and cells were indistinguishable from each other in Figure 3, prior studies indicate that the self‐alignment of the collagen extracellular matrix forms the framework for striated myofiber fusion and extension.

There was some deviation of myofiber alignment (Figure S2) across all TEMU variants, indicating alignment differences in the fabrication process. Nevertheless, the overall striated microarchitecture closely resembled that of native muscle tissue, characterized by fibrous and aligned morphology. The channel dimensions enabled collagen, myofiber, and axons to self‐align and bundle along the axis of the microtissue, in contrast to the more isotropic structure at the spherical endpoints.

### 3.3 Cross Section Image Demonstrates 3‐Dimensionality

A cross‐section of the region with the neuron aggregate (Figure 4A) was stained with immunofluorescence and imaged under confocal microscopy. Neuronal nuclei (NeuN, far red) were clustered in a subset of the non‐specific nuclei (Hoechst, blue). The phalloidin (green) stained for actin in both neurons and muscle however there is a clear separation in the neuron (Tuj1; β‐tubulin III, red) and muscle populations near the circumference, or outer region of the circular structure. Similarly, the longitudinal volumetric renderings of the confocal images (Figure 4B) provided a 3D view, demonstrating myocyte (α‐actinin, green) and motor neuron (NF200, red) self‐bundling.

**FIGURE 4:**
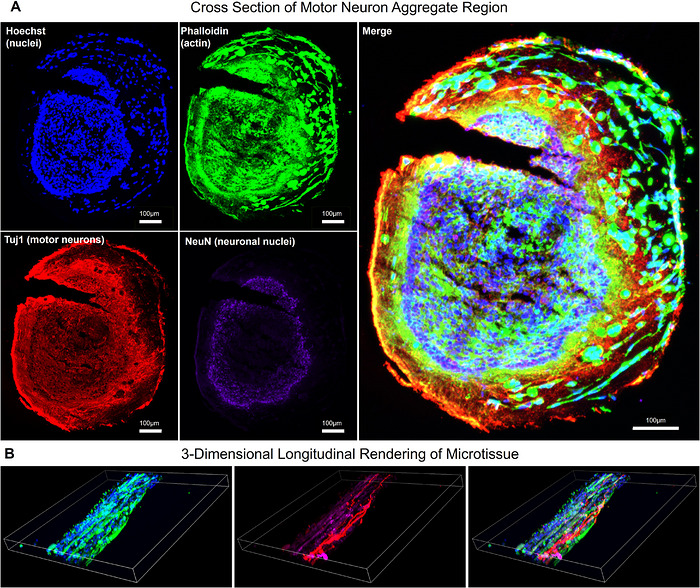
3D validation from cross section and volumetric rendering from confocal microscopy. (A) Representative cross section of a motor neuron aggregate. Immunofluorescent images of the axial section at the neuronal aggregate‐myofiber interface after 7 DIV in a TEMU‐uni. Scale bar is 100 µm for all images. The following were stained: nuclei (Hoechst, blue), actin (phalloidin, green), motor neurons (β‐tubulin III, red), and neuronal nuclei (NeuN, far red). (B) Representative immunofluorescent image of longitudinal volumetric rendering of a TEMU‐uni after 21 DIV. The following stains were nuclei (Hoechst, blue), actin (α‐actinin, green), motor neurons (NF200, red), and neuromuscular junctions (bungarotoxin, far red).

Together, the cross‐sectional and volumetric renderings confirmed the 3D organization of the microtissue. The spatial segregation between the neuronal nuclei and the Hoechst‐stained nuclei co‐labeled with actin highlighted the distinct localization of neuronal and myocyte populations. Although different cell types often co‐exist within most tissue niches, there remains an inherent separation of certain cell type populations to maintain tissue homeostasis [45]. Practically, this cross‐section specifically revealed the region of the motor neuron aggregate surrounded by ECM and myocytes, hence the highly dense spherical motor neuron center surrounded by isolated myocytes.

### 3.4 Long TEMUs To Indicate Scalability

Long TEMUs were fabricated using the same technique described previously (Figure 1) by modifying the length of the channels using CAD and subsequently 3D printing. The collagen ECM maintained its structure at 1 cm (Figure 5A), 2 cm (Figure 5B), 4 cm (Figure 5C1) paired with a zoomed in 500 × 500 µm volumetric rendering (Figure 5C2), and 8 cm (Figure 5D) paired with a phase image of the fully formed 8 cm TEMU (Figure 5E). The 3D rending shows respective myofiber and neuronal bundling (Figure 5C2). At a microscale, myocytes were aligned along the entire length of the fabricated microtissue (Figure 5Da–f). Of note, axons extended from the neuronal aggregates, in addition to some neurons migrating across the length of the microtissue.

**FIGURE 5:**
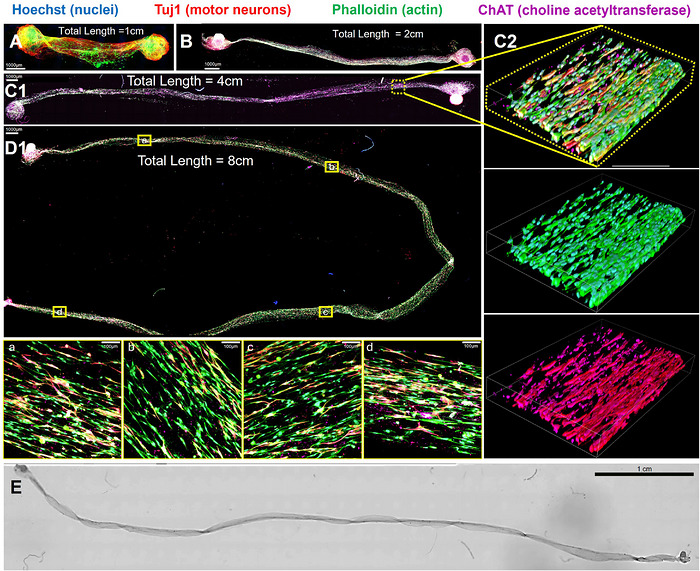
TEMUs can be cultured at varying lengths up to 8 cm, with no theoretical limit in length. (A) The default length of a TEMU was 1 cm. We observed cell survival in (B) 2 cm, (C1) 4 cm–with (C2) a 3D rendering of a 500 µm x 500 µm region of interest, and (D) 8 cm–(E) with phase imaging. Zoom‐in images of the 8 cm microtissue are shown as (a), (b), (c), and (d) and display a dissociated, nonspecific distribution of skeletal myocytes across the length of the microtissue. Scalebars are 1000 µm for (A)–(D). Scalebars are 100 µm for the zoom‐ins (a), (b), (c), and (d).

Certain muscular injuries, such as large muscle defects caused by trauma (i.e., bullet wounds) or cancer resections, are too complex for full recovery and severely impair motor capabilities, underscoring the need for clinical therapeutics to resolve function. Diminished muscle strength and mobility can lead to restricted limb motion, difficulty breathing or swallowing, pain, paralysis, and increased risk of falls.

A tissue‐engineered biologic therapeutic with the capacity for different lengths can lead to repair strategies for variable sized injuries. Injury size is unpredictable, therefore devising a repair strategy that can adapt with minimal lead‐time may reduce the time needed to address the problem. Although it is not clear how far axons extend from the aggregates in Figure 5, neuronal bodies appear to have migrated throughout the entire length. The length variability provides options for investigating cellular dynamics and addressing the role of myofiber length in functionality as well as disease progression.

### 3.5 Mitotic Inhibitor Influences Non‐Neuronal Cells

Recognizing a potentially prohibitive trophic factor in the original motor neuron media formulation, we investigated the effects of a mitotic inhibitor on engineered microtissues (Figure 6A). As described in the methods, the culture timeline was split into two phases: a myocyte growth phase (day 0–4 in culture) and a myocyte differentiation phase (day 4 to culture termination). Each phase required distinct media formulations, both paired with motor neuron media. Motor neuron media historically contained a mitotic inhibitor to limit excessive proliferation of glial cells in monocultures [32], however, this compound could also influence myocyte growth. To assess this possibility, we tested the effects of mitotic inhibitors in co‐culture conditions. The media formulations were defined as followed: (i) Media +/+, mitotic inhibitor present in both growth and differentiation phases, (ii) Media −/+, mitotic inhibitor absent during growth but present during differentiation phase, and (iii) Media −/−, mitotic inhibitor absent in both phases.

**FIGURE 6:**
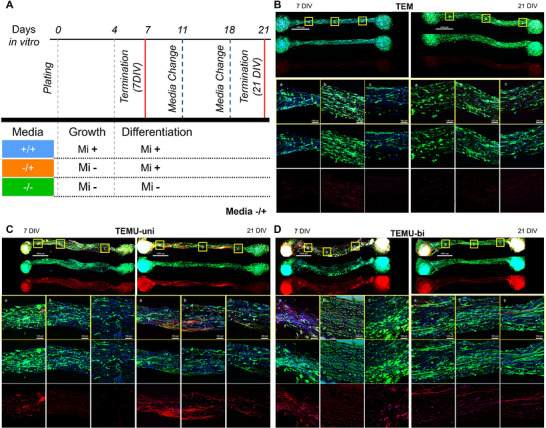
Conceptualization of the methods for investigating the role of mitotic inhibitors on myocyte growth and development. (A) Schematic detailing the protocol using different media compositions. Representative immunofluorescent images of engineered microtissues cultured with Media 2212/+ at both endpoints (a,c) and at a (b) mid‐section. The images show myocytes (α‐actinin, green), motor neurons (NF200, red), and nuclei (Hoechst) for the three cellular configurations (B) TEM, (C) TEMU‐uni, and (D) TEMU‐bi at 7 and 21 DIV. Scalebars are 1000 µm for whole length microtissues and 100 µm for zoomed in regions of interest.

Engineered microtissues were terminated at 7 days or 21 days in culture. To visualize microscale cell growth and morphologies, we examined the confocal microscopy using immunofluorescent staining. Myocytes were stained with α‐actinin (green), motor neurons were stained with NF200 (red), and nuclei was stained with Hoechst (blue) for three cellular configurations TEM (Figure 6B), TEMU‐uni (Figure 6C), and TEMU‐bi (Figure 6D) with all media variants; Media −/+ is shown as a representative image. For each microtissue, zoomed‐in images of the endpoints and the middle were taken and were shown (Figure 6B–D). As expected, neuronal markers were expressed in the two innervated configurations: TEMU‐uni and TEMU‐bi. Cells grew along the length of the engineered microtissue; myocytes scattered randomly whereas axons branched from the aggregates at the endpoints and extended along the ECM channel with time, as depicted most notably in the TEMU‐uni (Figure 6C).

Within the collagen ECM, myofibers and motor neuron axons self‐aligned, allowing quantification of myofiber morphology. The metrics were analyzed by media group and compared across cellular composition. When the mitotic inhibitor was present, myofiber length, aspect ratio, and fusion index increased over time, while width remained constant (Figure 7A). In the split condition, (Media −/+), myofibers also lengthened, exhibited increased aspect ratio and had greater fusion after 21 days compared to 7 days, with width remaining unchanged (Figure 7B). Notably, the increase in fusion from 7 days and 21 days in vitro was greater and more significant than in Media+/+. Similar trends were observed in Media −/− (Figure 7C).

**FIGURE 7:**
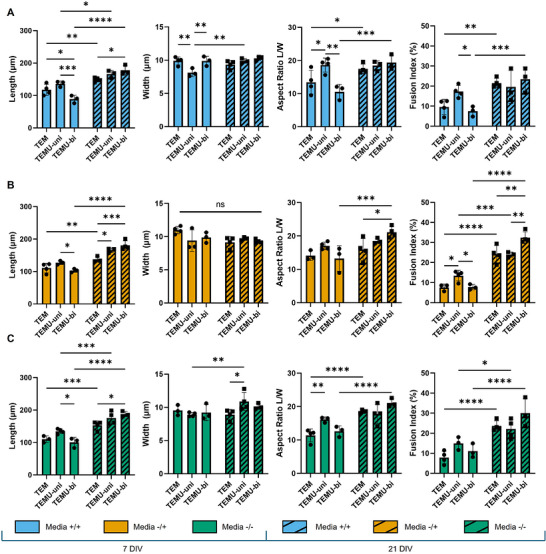
Structural parameters of skeletal myofibers grown in the tissue engineered muscle variants between three media compositions including (+) or excluding (2212) a mitotic inhibitor. Quantified measurements of skeletal myoblasts were recorded after 7 DIV and 14 DIV, both terminal timepoints. Myofiber length, width–or average thickness/diameter, aspect ratio, and fusion index were taken from (A) Media −/−, (B) Media −/+, and (C) Media +/+. Data were analyzed using a two‐way ANOVA that was run on all comparisons, specifically across time points and across TEMU variants. Significance was taken at *p* ≤ 0.05 (*), *p* ≤ 0.01 (**), *p* ≤ 0.001 (***), and *p* ≤ 0.0001 (****).

At 7 DIV, myofiber alignment showed no consistent pattern relative to innervation (Figure S2A). Significant differences in the myofiber alignment distribution were observed between TEMU‐bi and TEM groups in both Media −/+ and Media −/−, although the directionality differed: TEM showed a broader alignment in Media −/+ but a narrower one in Media −/−. Likewise, across the media groups, alignment distribution differed significantly between Media −/− and Media +/+ within the TEM configuration. For TEMU‐bi, Media +/+ and −/− had broader alignment distributions than −/+. Within Media −/+ at 7 DIV, the apparent difference between TEM and TEMU‐uni was not statistically significant (Kolmogorov‐Smirnov Test, the p‐value is 0.08). For the 21 DIV groups, innervation exerted a clear difference in myofiber angle distribution (Figure S2B). In Media +/+ and −/−, TEMU‐bi displayed broader alignment than TEM, whereas the opposite pattern was observed in −/+ media.  Across media groups, the myofibers have a tighter alignment in Media +/+ and myofibers have a broader alignment in TEMU‐bi in the Media −/− group.

Interestingly, TEMU‐uni were relatively consistent across all media groups and time points, although myofiber alignment distribution significantly differed in TEMU‐unis across time points for all media groups (Figure S2C). In the media lacking the mitotic inhibitor during any phase, myofiber alignment became more narrowly distributed at 21 DIV than at 7 DIV consistently for TEMU‐uni. The opposite effect–the distribution of myofiber angle increased over time–was seen in Media +/+. Much of the myofiber angle distribution data informs how robust the engineered microtissue is as a biomimetic tool. Microtissues that had a tighter myofiber angle distribution indicated anisotropic uniformity in arrangement and therefore consistent with mature skeletal muscle.

Individual myofiber morphologies were measured to determine myofiber growth and development. Myofibers are longer at 21 DIV than at 7 DIV (Figure 7) and for which innervation further lengthens the myofibers. The width of myofibers remains consistent across cell configurations and over time, indicating anisotropic growth (Figure 7). The aspect ratio (Figure 7) mirrors the myofiber lengths, given that myofiber width remained statistically similar from one another at 7 DIV. This suggests that myofiber width did not change significantly at rest–that is, without hypertrophy or applied mechanical stress or strain. At 21 DIV, innervation played a role in increasing the aspect ratio. Furthermore, myofibers fuse more overtime with increased fusion index between the 7 DIV and 21 DIV time points (Figure 7). Only the TEMU‐bi demonstrated greater fusion than the TEM and TEMU‐uni groups after 21 DIV. The innervation paradigm did not yield a consistent preference for one or two neuron populations within the 21‐day timeframe.

Additionally, the presence of a mitotic inhibitor did not significantly affect myocyte morphological parameters, with or without innervation (Figure S3). Differences became apparent only when considering sarcomere organization and neuromuscular junction formation.

### 3.6 The Presence of Sarcomeres Measures Maturity and Sarcomere Length

Sarcomeres were distinctly seen in cultures after 21 DIV (Figure 8A). To visualize the sarcomeres, we examined the confocal microscopy images at a higher magnification than in Figure 6 and measured both the percentage of myofibers with sarcomeres in each microtissue (Figure 8B) and the distance between adjacent sarcomeres (Figure 8C). The proportion of myofibers containing sarcomeres varied with media composition, although differences were not statistically significant (Figure 8B). In the absence of mitotic inhibitors in the media, Media −/−, innervation increases the percentage of myofibers with sarcomeres, although only statistically significant comparing the non‐innervated TEM with the single motor neuron aggregate TEMU‐uni. Overall, innervation tended to increase sarcomere formation, but the only significant difference was as noted above. No sarcomeres were observed in the TEMs cultured in Media −/+, precluding measurement of sarcomere spacing for that group. The spread of between‐sarcomere lengths is uniform across all cell configurations and media compositions, most lengths ranging between 1 and 4 µm with a few spaced outliers (Figure 8D). The sarcomere spacing remains consistent with previously reported values [35].

**FIGURE 8:**
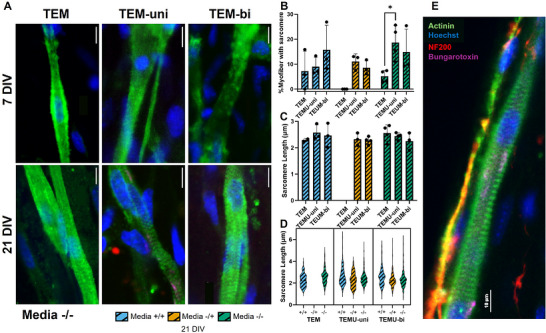
Sarcomeres were present in myofibers cultured in the engineered microtissues. (A). Representative immunofluorescent images of individual myofibers containing sarcomeres in engineered microtissues with Media −/−, or no mitotic inhibitor. The images show myocytes (alpha‐actinin, green) and nuclei (Hoechst) for the three cellular configurations TEM, TEMU‐uni, and TEMU‐bi 7 and 21 DIV. Scalebars are 10 µm. (B) The percentage of myofibers with sarcomeres out of the total sampled myofibers in each microtissue and cell configuration. (C) Sarcomere length across media and cellular compositions. (D) Violin plots of the sarcomere length as summarized in (C). (E) Representative image of neuromuscular junctions (far red, bungarotoxin) co‐labeled on a mature myofiber (green, α‐actinin) alongside neurons (red, NF200). Two‐way ANOVA were run on all comparisons (*p < 0.05); there were no data for TEM, −/+.

It is notable that sarcomeres were absent in TEMs cultured with Media −/+, despite being present in Media −/− and Media +/+. Sarcomere maturation occurs during the post‐mitotic cell‐fusion phase [46]. If the mitotic inhibitor was fully effective, myocyte proliferation might be completely suppressed, leaving only the original plated cells available to fuse.  Although cell proliferation was not directly measured, existing cells retained the capacity to fuse and form sarcomeres under any condition. During the proliferative phase in embryonic cardiomyocytes, sarcomeres disassemble only to reassemble fully during cytokinesis [47]; skeletal myocytes may undergo the same mechanism. This complexity may explain why skeletal myocytes generally do not divide postnatally [47] unless during regeneration with satellite cells located beneath the basal lamina [48, 49, 50].

### 3.7 Neuromuscular Junctions and Contractility Demonstrate Functionality

To evaluate formation of functional NMJs within the engineered microtissue, we examined bungarotoxin immunofluorescence in the innervated engineered microtissues at 21 DIV (Figure 9A,B). NMJs connect motor endplates to skeletal muscle and enable neuronal activation of muscle contraction [51]. The presence and morphology of NMJs therefore serve as a key indicator of functional innervation. Positive bungarotoxin staining identified self‐formed, developing NMJs within the microtissues, confirming receptor clustering at neuron–muscle contact points. Mature NMJs typically exhibit a complex, pretzel‐like morphology; by comparison, the NMJs–acetyl‐choline receptor expression–observed in the TEMU‐uni constructs appeared less organized, similar to those seen in postnatal muscle [52, 53]. These findings suggest that NMJ formation within the TEMU platform follows an in vitro maturation trajectory resembling early developmental stages.

**FIGURE 9:**
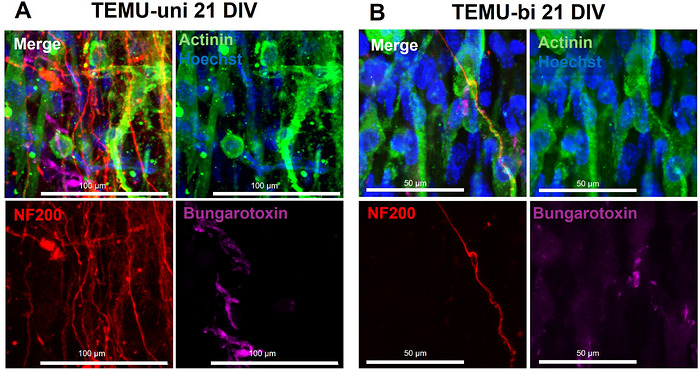
Representative immunofluorescent images of neuromuscular junctions (NMJ) in experimental in vitro conditions. (A) Mature‐appearing NMJ were found and imaged in a TEMU‐uni microtissue after 21 DIV. Scalebars are 100 µm. (B) NMJ were also found in TEMU‐bi microtissue after 21 DIV. Scalebars are 50 µm.

To assess neuromuscular functionality, innervated and non‐innervated microtissues were exposed to 1 mM glutamate for one hour. Glutamate, a presynaptic neurotransmitter, evoked rhythmic skeletal‐muscle twitching in a subset of the innervated constructs, while non‐innervated microtissues remained quiescent (Video S1). Data was gathered by tracking the displacement of specific locations on contracting myofibers over time (Figure 10A,B). Myofiber contractions were observed at a rate of 1.48 ± 0.04 contractions/second with a mean maximum displacement of 4.88 ± 0.70 µm and average displacement of 1.10 ± 0.20 µm longitudinally across myofibers in the innervated group. Representative curves show contraction displacement and velocity (Figure 10C,D respectively). Non‐innervated groups exhibited no myofiber contractions and therefore yielded no quantifiable data. Since glutamate would stimulate neuronal activation but not the myofibers directly, these data showing glutamate‐induced contractions indicated direct synaptic coupling between neurons and myofibers. These findings serve as proof‐of‐concept that the TEMU system can support functional NMJ formation and neuromuscular signaling, and demonstrate the feasibility of a more quantitative, dynamic assessment in future studies.

**FIGURE 10:**
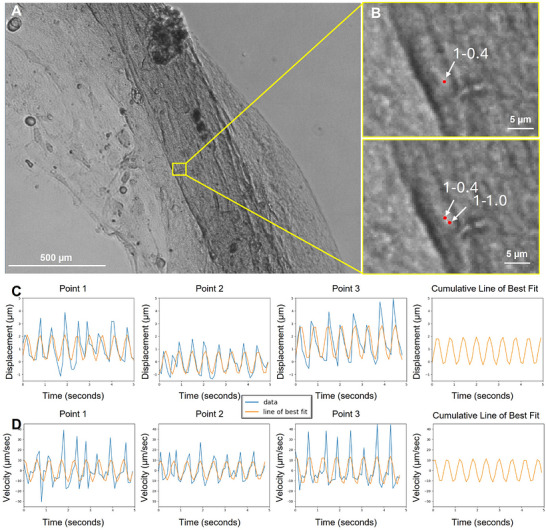
Quantified measures of muscle contraction. (A) Phase microscopy of muscle contraction in an innervated microtissue after 1 h incubation in 1 mM glutamate (scale bar = 500 µm) with (B) zoom‐ins indicate the change in position of the same point on the myofiber taken in video at 0.4 sec and 1.0 sec (scale bar = 5 µm). (C) Displacement and (D) velocity graphs of muscle contraction were derived from quantification and paired with a sinusoidal line of best fit for each dataset. Data were taken from three points of the contracting myofiber, also shown in Video S1. The far‐right panels of (C) and (D) are the averaged data of the regression lines.

Together, these findings demonstrate that NMJs within the TEMU platform can self‐assemble and generate functional output in a 3D co‐culture system. While the present study focused on establishing NMJ formation and functional innervation, quantitative characterization of synaptic organization, including NMJ density, area, and colocalization metrics, was not performed and represents an important avenue for future investigation. Future studies will quantify contractile force as a function of neuronal population density, stimulation modality (chemical vs. optogenetic), and culture duration, alongside measurements of calcium transient amplitude and detailed morphological analyses of NMJ organization to further validate and characterize functional NMJ formation.

### 3.8 Limitations

This study establishes a scalable platform for generating pre‐innervated 3D skeletal muscle bundles; however, several limitations of the in vitro model should be acknowledged. The microtissues represent a simplified approximation of a native motor unit since they are composed solely of C2C12 myoblasts and embryonic rat motor neurons. Incorporating cell types, such as Schwann cells or endothelial cells, could enhance ECM remodeling, axonal guidance, and NMJ maturity. In addition, while the collagen hydrogel scaffold effectively promotes alignment, it does not fully replicate the stiffness or biochemical complexity of native muscle ECM, which may result in myofibers that differ from endogenous muscle bundles.

Characterization of neuromuscular integration was primarily based on immunocytochemistry and exploratory glutamate‐evoked responses. Future work will include a more comprehensive quantification of NMJ density, co‐localization, and pre‐ and post‐synaptic marker expression, along with gene and protein expression profiling, to provide a more complete assessment of tissue maturation. Similarly, additional functional assays assessing contractile force generation, fatigue characteristics, and electrophysiological activity would further strengthen the physiological characterization of the model.

Construct reproducibility also varied, with approximately 20%–30% collapsing during or after culture, suggesting fabrication sensitivity to ECM formulation, cell density, and handling. Finally, the media optimization study focused on a single mitotic inhibitor, leaving the influence of other growth factors unexplored.

Taken together, these factors define the current TEMU system as a controlled yet foundational in vitro platform. Addressing these constraints through the inclusion of supportive cell populations, more physiological ECMs, and quantitative functional assays will improve the model and facilitate its eventual translation for in vivo applications.

### 3.9 Emerging Regenerative Strategies for VML

There are 3D voluminous tissue engineered muscles fabricated in ECM [7, 13, 16, 17, 54, 55] that form thick myofibers with well‐defined sarcomeres after 14 DIV. Most methods use pillars to anchor the microtissues and prevent them from fully collapsing in on themselves compared to other methods which involve free‐floating bulbous ECM [56]. Our fabrication technique takes advantage of topography with an indented reservoir which provides the microtissue with a self‐anchoring shape while still retaining its removability. There are some 3D printed tissue‐engineered muscles that can be implanted and result in biomimetic structures [57], however our smaller engineered muscle bundles have physical flexibility and can be joined with additional microtissues. Indeed, by leveraging both the self‐anchoring and the structural integrity of confined channels, the TEMU fabrication can be extended up to at least 8 cm. Subsequently, the individual TEMUs can be removed without damage from culture and several TEMUs can be bundled together in parallel to create macro‐scale engineered muscle. In other notable approaches, microfluidic chips provide an exterior, confined scaffolding for ECM and myocytes to align in [41] over otherwise random 2D configurations [9]. These and other techniques often show self‐alignment and spontaneous contraction [13, 16, 54, 58, 59]. Compared to our previously developed engineered microtissues cultured on electrospun nanofiber sheets [21], the extracellular matrix‐based TEMU platform provides a more economical, biomimetic, and modular approach to muscle and axon bundling. The transparent ECM environment also enables direct visualization of cell health and contractile behavior relative to the opaque polycaprolactone nanofiber substrates. Although neuromuscular junction density was not quantified, TEMUs exhibited morphologically mature NMJs, in contrast to the embryonic phenotypes observed in nanofiber sheet cultures. However, unlike other models, our fabrication technique results in a self‐contained, removable, and innervated 3D translational muscle transplant.

### 3.10 Future Directions

The TEMU technology built and developed in this study offers numerous avenues of investigation going forward. Previous studies have successfully engineered muscle units with robust myofiber formation and maturation [7, 54, 55]. However, few have achieved increases in the overall volume and size of implantable tissue. The resizable microtissue unit described here has the capacity to bulk up and fill large, human‐sized muscle defects [60]. Although cell dose, response to electrical stimulation, response latency, fatigue resistance, and generated force measurements were not incorporated in the current study, their inclusion will be essential to further validate the functional performance of this engineered skeletal muscle model. Other groups have shown optogenetically programmed innervation to activate muscle contraction [13, 16]. Building on this, future studies should employ optogenetically programmable neurons to create a biohybrid interface [30, 61, 62], further enhancing the TEMU platform's ability to control bulk muscle contraction. Additional research should explore the mechanisms of reflex pathways and monitor calcium transients upon stimulation using sensory neurons, motor neurons, and skeletal muscle. Another promising direction would be to adapt tissue culture to human iPSCs, moving closer to a clinically relevant therapeutic application.

## 4 Conclusions

We present methodology for the biofabrication of centimeter‐scale innervated 3D bundled myofibers, termed Tissue Engineered Motor Units (TEMUs) that replicated in vivo architecture. These constructs consist of self‐assembled, aligned myofiber bundles embedded within a 3D collagen hydrogel and innervated by axons projecting from discrete population(s) of spinal motor neurons. During development, myofibers exhibit anisotropic elongation and progressive fusion over time. While the presence of innervation did not overtly affect developing myocyte morphology in the tissue engineered muscle developed in this study, the presence of aggregated motor neurons and axonal integration enhanced sarcomere formation and myofiber contractility compared to non‐innervated controls. Indeed, innervation enabled skeletal muscle development as shown by the robust presence of sarcomeres and functional neuromuscular junctions. Importantly, TEMU biofabrication was scalable, enabling the generation of modular myofiber bundles up to at least 8 cm in length and the assembly of large‐scale macro‐bundles. This work extends previous efforts to engineer muscle and axonal tissues within guided 3D architecture.

The resulting innervated engineered muscle tissue provides a 3D biofidelic in vitro platform to study the critical role of innervation in muscle development, function, and injury/disease. Moreover, this holds potential as an implantable composite tissue to facilitate muscle replacement by promoting re‐innervation and functional recovery of host and graft muscle after severe trauma. Our novel contributions include the successful 3D co‐culture of motor neurons and myofibers, flexibility of cell sourcing, and demonstration of scalable biofabrication. These proof‐of‐concept studies establish a foundation for advancing 3D innervated myofiber platforms as both a bio‐fidelic model system and implantable therapeutic tissues.

## Author Contributions

M.C.H. S.D. and D.K.C. conceived of the approach. M.C.H. designed and carried out experiments and analyzed data. M.H. wrote the first draft of the manuscript, with input from E.K., S.D., F.M., and D.K.C.

## Funding

Financial support was provided by Department of Health at the Commonwealth of PA [Health Research Formula Fund Grant (Cullen)], the US Department of Defense [HT9425‐24‐1‐0804 (Cullen and Das)], and the National Institutes of Health [R01‐AR083489 (Cullen) and R01‐NS117757 (Cullen)]. Any opinion, findings, and conclusions or recommendations expressed in this material are those of the authors(s) and do not necessarily reflect the views of the Commonwealth of PA, Department of Defense, or National Institutes of Health.

## Ethics Statement

These studies were done in accordance with approvals from the Institutional Animal Care and Use Committee (IACUC) at the University of Pennsylvania and the CMC VA Medical Center in Philadelphia.

## Conflicts of Interest

U.S. Patent Application 17/291,869 and PCT International Patent Application PCT/US2019/060585 titled “Engineering of Innervated Tissue and Modulation of Peripheral Organ Activity” (DKC, SD) are related to the techniques described in this paper. The other authors declare that they have no competing interests.

## Supporting information




**Supporting File 1**: mabi70236‐sup‐0001‐SuppMat.docx.

**Supporting File 2**: mabi70236‐sup‐0002‐FigureS1‐S3.zip.

**Supporting File 3**: mabi70236‐sup‐0003‐VideoS1.pptx.

## Data Availability

The raw data supporting the conclusions of this article will be made available by the authors, without undue reservation.
