# Supplementary material for: Modular Tissue‐Engineered Motor Units Featuring Spinal Motor Neurons Innervating Self‐Assembled Myofiber Bundles
Source: Macromol Biosci. 2026 Aug 13;26(8):e00660. doi: 10.1002/mabi.202500660 (PMC13471878; doi:10.1002/mabi.202500660)
Supplement: Supplementary file 2 [file MABI-26-e00660-s003.docx]

**Supplementary Figure 1.** Design iterations of the 3D printed scaffold to form PDMS channels for cell/ECM plating. (A) Version 1 was the progression of channel designs and considerations for 12-well plate wells. The channels were made with respect to separating cell population while retaining the integrity of one whole microtissue. Version 2 was the refinement of dimensions for 6-well plate wells, ultimately choosing Version 2.1 as the final design. (B) Blueprints of the long microtissues that are 1 cm, 2 cm, 4 cm, and 8 cm. (C) The specified dimensions of the 3D printed scaffolds indicate the channel size and the resulting PDMS mold.

**Supplementary Figure 2.** Histograms of myofiber angle alignment to investigate uniformity in myofiber directionality. (A) Myofiber angle distributions of cultures after 7 DIV. (B) Myofiber angle distributions of cultures after 21 DIV. (C) Simplified illustration of statistical differences between histograms across timepoints. Arrows indicate statistical significance with * notating differences across TEMU variants within media groups, # notating differences across media groups, and + notating TEMU variants across timepoints within media groups. (D) Representative image of how myofiber angle alignment was measured and recorded with the yellow measurement line in reference to the dashed white line. A Kolmogorov-Smirnov test was run on all comparisons (*p<0.05, **p<0.001, ***p<0.0001, ****p<0.00001).

**Supplementary Figure 3.** The same data from Figure 7 except rearranged to visualize the structural parameters of skeletal myofibers grouped together. As such, quantified measurements of skeletal myoblasts were recorded after 7 DIV and 14 DIV including average myofiber (A) length, (B) myofiber width – or thickness/diameter, (C) aspect ratio, and (D) fusion index were taken from 3 media compositions including (+) or excluding (-) a mitotic inhibitor: Media -/-, Media -/+, and Media +/+. Data were analyzed using a two-way ANOVA that was run on all comparisons, specifically across time points and across media composition. Significance was taken at p≤0.05 (*), p≤0.01 (**), p≤0.001 (***), and p≤0.0001 (****).

**Supplementary Figure 4. Quantification of chemically stimulated myofiber contraction.** (A) Phase microscopy video indicating evidence of functional innervation. TEMU neurons were directly stimulated via addition of glutamate [1mM], which in turn activates muscle contraction via neuromuscular innervation**.**
